# Novel three-dimensional *in vitro* models of ovarian endometriosis

**DOI:** 10.1186/1757-2215-7-17

**Published:** 2014-02-06

**Authors:** Doerthe Brueggmann, Claire Templeman, Anna Starzinski-Powitz, Nagesh P Rao, Simon A Gayther, Kate Lawrenson

**Affiliations:** 1 Department of Obstetrics and Gynecology, University of Southern California/Keck School of Medicine, IRD, 2020 Zonal Avenue, Los Angeles, California 90033, USA; 2 Department of Cellbiology and Neuroscience, Goethe University, Max-von-Laue-Straße 13, Frankfurt am Main 60438, Germany; 3 Pathology and Lab Medicine, David Geffen University of California Los Angeles, 22-26 Rehab Cntr, 1000 Veteran Ave, Los Angeles, California 90024, USA; 4 Department of Preventive Medicine, University of Southern California/Keck School of Medicine, Harlyne Norris Research Tower, NRT2517G, 1450 Biggy Street, Los Angeles, California 90033, USA

**Keywords:** Ovary, Endometriosis, Cell culture, Three-dimensional *in vitro* modeling, Real-time semi-quantitative PCR, RNA sequencing

## Abstract

**Background:**

Endometriosis is characterized by the presence of functional endometrial tissue outside of the uterine cavity. It affects 1 in 10 women of reproductive age. This chronic condition commonly leads to consequences such as pelvic pain, dysmenorrhea, infertility and an elevated risk of epithelial ovarian cancer. Despite the prevalence of endometriosis and its impact on women’s lives, there are relatively few *in vitro* and *in vivo* models available for studying the complex disease biology, pathophysiology, and for use in the preclinical development of novel therapies. The goal of this study was to develop a novel three-dimensional (3D) cell culture model of ovarian endometriosis and to test whether it is more reflective of endometriosis biology than traditional two dimensional (2D) monolayer cultures.

**Methods:**

A novel ovarian endometriosis epithelial cell line (EEC16) was isolated from a 34-year old female with severe endometriosis. After characterization of cells using *in vitro* assays, western blotting and RNA-sequencing, this cell line and a second, already well characterized endometriosis cell line, EEC12Z, were established as *in vitro* 3D spheroid models. We compared biological features of 3D spheroids to 2D cultures and human endometriosis lesions using immunohistochemistry and real-time semi-quantitative PCR.

**Results:**

In comparison to normal ovarian epithelial cells, EEC16 displayed features of neoplastic transformation in *in vitro* assays. When cultured in 3D, EEC16 and EEC12Z showed differential expression of endometriosis-associated genes compared to 2D monolayer cultures, and more closely mimicked the molecular and histological features of human endometriosis lesions.

**Conclusions:**

To our knowledge, this represents the first report of an *in vitro* spheroid model of endometriosis. 3D endometriosis models represent valuable experimental tools for studying EEC biology and the development of novel therapeutic approaches.

## Background

Endometriosis is a chronic condition affecting around 10% of reproductive age women [1]. Typically, functional endometrial tissue is present outside the uterine cavity and results in symptoms that include pelvic pain, dysmenorrhea and dyspareunia [2]. Endometriosis patients often experience infertility and are at an elevated risk of epithelial ovarian cancer [2,3]. Endometriosis can be classified into three subtypes: lesions in the pelvic peritoneum; ovarian endometriosis that may occur as superficial lesions on the surface of the ovary or as cysts lined with endometrioid epithelium (endometrioma); and deep-infiltrating lesions of the rectovaginal septum [2,4]. Ovarian endometriosis is of particular interest, as a proportion of ovarian cancers arise from ovarian endometriotic lesions, particularly clear cell and endometrioid ovarian carcinomas [5,6].

Despite the prevalence of endometriosis and its significant impact on women’s lives, there are relatively few *in vitro* and *in vivo* models available for studying the complex disease biology, pathophysiology, and for use in the preclinical development of novel therapies. One widely used *in vitro* model comprises cell lines of epithelial (EEC12Z) and stromal (ESC22B) origin harvested from peritoneal endometriosis lesions [7-9]. Co-injection of these cells into mice results in peritoneal lesions that recreate histological features of human endometriosis *in vivo*[8]. This particular model has been used to study various aspects of endometriosis cell biology including hormone signaling, cell-cell adhesions, as well as to conduct candidate gene studies [9-11]. However, this model was established from a peritoneal lesion, and there is a real need for additional models that mimic other subtypes of this disease in order to better understand the pathophysiology of endometriosis subtypes, and for the development of new treatment strategies.

A major limitation of existing *in vitro* models of endometriosis is that they have been established by culturing endometriosis epithelial cells (EECs) as monolayers on tissue culture plastics (i.e. as two-dimensional (2D) cultures). *In vivo*, EECs exist within a dynamic three-dimensional (3D) microenvironment and constantly interact with a stroma containing immune cells, fibroblasts, vasculature and a heterogeneous network of extracellular matrix. Endometriosis cells *in vivo* also form cell-cell interactions through the entire cell surface. By contrast, cells in 2D can only interact along a small proportion of the plasma membrane. Several studies have now reported on improvements in *in vitro* modeling of several diseases when target cells are cultured as 3D models; cultured cells maintained in 3D resemble the tissues of origin more closely than the same cells cultured in 2D [12-14]. However, to our knowledge, there are no studies reporting *in vitro* spheroid models of endometriosis. Such models could be particularly useful for developing novel therapies for this disease [15] and for studying the links between endometriosis and ovarian cancer.

To better model the biology of ovarian surface endometriosis, we have established and characterized a novel endometriosis epithelial cell (EEC) line, EEC16, from a 34-year old patient diagnosed with ovarian endometriosis. EEC16 and a second endometriosis cell line (EEC12Z) from a patient with peritoneal endometriosis were established as *in vitro* 3D cell culture models and the morphological and molecular features evaluated. EECs grown as 3D cultures mimic endometriosis lesions *in vivo* more closely than 2D cultured counterparts, suggesting that these models are robust representations of human endometriosis for future use in understanding the etiology of endometriosis and identifying novel therapeutic targets for the disease.

## Methods

### Primary tissue collection

Endometriosis cells (EEC16) were isolated from a 34-year pre-menopausal patient with severe, histologically confirmed endometriosis. EEC16 cells were collected from a superficial endometriosis lesion on the surface of the ovary. The ovary of the patient was removed at surgery and the ovarian surface brushed with a sterile cytobrush that was then placed into 7 mls culture medium and agitated to release the cells.

Normal ovarian epithelial cells (OSEC10, OSEC181 and OSEC11) were obtained from women undergoing gynecological surgery for conditions that did not involve the ovaries (endometrial carcinoma or hyperplasia). Cells were collected by brushing the ovaries with a sterile cytobrush, as described above. Ovaries were confirmed to be free of disease by histopathological assessment. All OSECs used in this study are morphologically and phenotypically similar and are representative of the ~80 OSEC cell lines we have characterized in our laboratory. The cell-containing medium was transported to the tissue culture laboratory and transferred to a 25 cm^2^ tissue culture flask. Cell growth was monitored by phase microscopy, and cells were fed twice weekly. Once cells reached 80% confluency, the culture was passaged.

For histology and real-time PCR experiments, tissue samples were obtained from patients undergoing laparoscopy at Keck Hospital of USC for endometriosis or other benign gynecological conditions. Biopsy material was transferred in either RPMI media (Sigma-Aldrich, United States) or RNAlater (Ambion, United States) and stored at −80°C.

### Cell culture

Endometriosis epithelial cells (EEC16) and OSECs were maintained in NOSECM [16]: MCDB105:Medium 199 (mixed in a 1:1 ratio) supplemented with 15% fetal bovine serum (FBS, Hyclone, United States), 10 ng/ml epidermal growth factor, 0.5 mg/ml hydrocortisone, 5 mg/ml insulin, and 34 mg protein/ml bovine pituitary extract, (all Sigma, United States) plus penicillin/streptomycin (Mediatech, United States). SV40 transformed endometriosis epithelial cells (EEC12Z) [17] were cultured in Dulbecco’s Minimal Essential Medium supplemented with 10% fetal bovine serum (FBS, PAA Laboratories, Austria) and penicillin/streptomycin (Mediatech, United States). Control cells for anchorage-independent growth assays and Western blotting (MCF7, MDA-MB-231, IGROV and SKOV 3) were grown in the media recommended by ATCC or The Lawrence Berkeley National Laboratory [18]. All cell lines used in this study were routinely tested for mycoplasma infection.

### EEC16 *in vitro* characterization

To perform Western blot analysis of marker expression, cells were harvested at 80% confluency, were washed twice in phosphate buffered saline (PBS) and then lysed using Triton-X lysis buffer (20 mM Tris-Cl pH 7.5, 150 mM NaCl, 1% Triton-X 100, 1.2 μg/ml aproptinin, 100 μg/ml leupeptin, and 1 mM phenylmethylsulfonyl fluoride, all purchased from Sigma, United States). Lysates were rotated at 4°C for 30 mins before clearing insoluble proteins by centrifugation for 10 mins at 4°C at 14000 rpm. Protein concentrations were determined using the Coomassie Plus Protein Assay (Thermo Scientific, United States), according to manufacturer’s instructions. 5–10 μg protein was denatured and separated using SDS-polyacrylamide gel electrophoresis. Proteins were transferred onto polyvinylidene fluoride membranes overnight, and probed using standard protocols. The antibodies used were pan-cytokeratin (sc-8018, Santa Cruz, United States), vimentin (clone VIM 3B4, Millipore, United States), Estrogen Receptor α (ERα, sc-543, Santa Cruz, United States), E-Cadherin (clone EP700Y, Millipore, United States), P-Cadherin (clone 12H6, Invitrogen, United States), N-Cadherin (clone 3B9, Invitrogen, United States) and β-actin (A5060, Sigma, United States). All primary antibodies were used at a 1:1,000 dilution except for anti-E-Cadherin, which was used at a 1:5,000 dilution.

Chromosomal analyses and karyotyping were performed at Pathology and Lab Medicine, David Geffen University of California Los Angeles. Cells were plated into a 25 cm^2^ flask and harvested when subconfluent. Cells were Giemsa-banded following routine cytogenetic methods. Twenty metaphase cells were analyzed and karyotyped under a Zeiss bright field microscope (Zeiss, Germany) equipped with image analysis hardware and software.

To perform growth curves, 1 × 10^5^ cells were plated in triplicate. Cultures were passaged when they reached 80% confluency and population doublings (PD) were calculated using the following formula:

PD = log (total cell number on day N/initial cell number)/log2.

For migration and invasion assays, cells were starved for 24 hours, and applied to cell permeable transwell inserts, in triplicate. For migration assays, 3 × 10^4^ cells were applied to migration inserts (Greiner Bio One, Austria); for invasion assays, 0.125 × 10^6^ cells were applied to rehydrated QCM ECMatrix invasion chambers (Millipore, United States). 10% FBS was used as a chemoattractant. After 24 hours, remaining cells were removed from the upper chamber of the inserts. To quantify migration, membranes were fixed in 100% methanol (VWR, United States), stained with crystal violet solution (Sigma, United States; 5 mg/ml in 2% ethanol (VWR, United States) and cells were counted by brightfield microscopy. Fluorimetric quantification of invaded cells was performed according to the manufacturer’s instructions. Anchorage independent growth assays were performed by plating 2 × 10^4^ cells in culture medium containing 0.3% Noble Agar (Sigma, United States) over a base layer of complete medium containing 0.6% Noble Agar. Five replicates were plated for each cell line; SKOV3 ovarian cancer cells served as a positive control. After 4 weeks, cells were fixed and stained with 1% p-iodonitrotetrazolium violet (Sigma, United States) in 100% methanol (VWR, United States). Colonies were counted using phase microscopy. To test for phenotypic differences between cell lines, two-tailed unpaired Student’s T-tests were used.

### RNA-sequencing analysis and gene ontology analyses

RNA was extracted from EEC16 and OSEC11 cultures using the Illustra RNAspin mini kit with on column DNase treatment (GE Healthcare, United States) according to manufacturer’s guidelines. RNA-sequencing was performed at the USC Epigenome Core Facility. Briefly, RNA samples were quality checked (QC’d) using the Agilent Bioanalyzer (Agilent Technologies, United States) and polyA RNA-seq cDNA libraries prepared the TruSeq™ RNA sample prep kit (Illumina, United States). Libraries were barcoded and 4 samples multiplexed per lane for sequencing on the Illumina HiSeq™ 2000 using 50 bp paired-end reads. Data were exported, QC’d and analysed using SimBiot software. QC’d data were mapped to the genome using TopHat, normalized gene expression quantified using Cufflinks and differential expression analyses performed using CuffDiff. Gene ontology (GO) analyses were performed using DAVID (http://david.abcc.ncifcrf.gov/) for all genes significantly differentially expressed between EEC16 and OSEC11 after adjustment for multiple testing (q value < 0.05). GO terms with a Benjamini-adjusted p-value < 0.05 were considered to be significantly enriched for in this dataset. RNA-seq data have been deposited onto the Gene Expression Omnibus (http://www.ncbi.nlm.nih.gov/geo/, GEO number to be confirmed by time of publication).

### Three-dimensional cell culture, histology and immunohistochemistry

Cell culture plastics (diameter: 100 mm) were twice coated with 1.5% polyHEMA (Sigma, United States) dissolved in 95% ethanol (VWR, United States). Coated plates were allowed to dry completely before use. Coated plates were washed for ~5 mins with 1× PBS and 1–3 × 10^6^ cells were added in a final culture volume of 20 mls. Cultures were fed twice weekly before processing into paraffin or RNA extraction. The diameter of the spheroids was assessed by brightfield microscopy.

For paraffin embedding, human endometriosis tissue and spheroids were fixed in neutral buffered formalin (30 mins, at room temperature), washed and transferred into 70% ethanol. The samples were processed into paraffin, sectioned and stained with H&E at the USC Surgical Pathology Laboratory. Immunohistochemical staining was performed at the USC Department of Pathology Immunohistochemistry Laboratory.

### RNA extraction and gene expression analysis

RNA was extracted from 2D and 3D cultured cells and human endometriosis tissue samples as described above; after mechanical disruption, samples were lysed using 350 μl RA1 lysis buffer (containing 1% β-mercaptoethanol). Samples were quantified and reverse-transcribed using qScript and random hexamer primers (Quanta Biosciences, United States). The final PCR mixture contained 0.5 μl each of forward and reverse primers (final concentration of 100–500 nM, primer sequences in Additional file 1: Table S1), 12.5 μl 2× SYBR PCR mix (Quanta Biosciences, United States), and 1 μl cDNA. Using an ABI 7900HT Fast Real-Time PCR system (Applied Biosystems, United States), the samples were run using the following conditions: 2 mins at 50°C, 10 mins at 95°C, 40 cycles of 15 secs at 95°C, and 1 min at 60°C. Data were standardized in relation to the house-keeping gene *GAPDH* and analyzed using the ΔΔCt relative quantification method. To compare changes in gene expression in 2D and 3D, two-tailed paired Student’s T-tests were performed.

### Ethical approval

For primary cell culture, tissues were collected, with informed consent, under the approval of the University College London/University College London Hospitals UCL/UCLH Ethics Committee. The collection of endometriosis tissue for real-time PCR experiments was approved by the USC Institutional Review Board.

## Results

### Establishing a novel *in vitro* model of endometriosis epithelial cells

**Figure 1:**
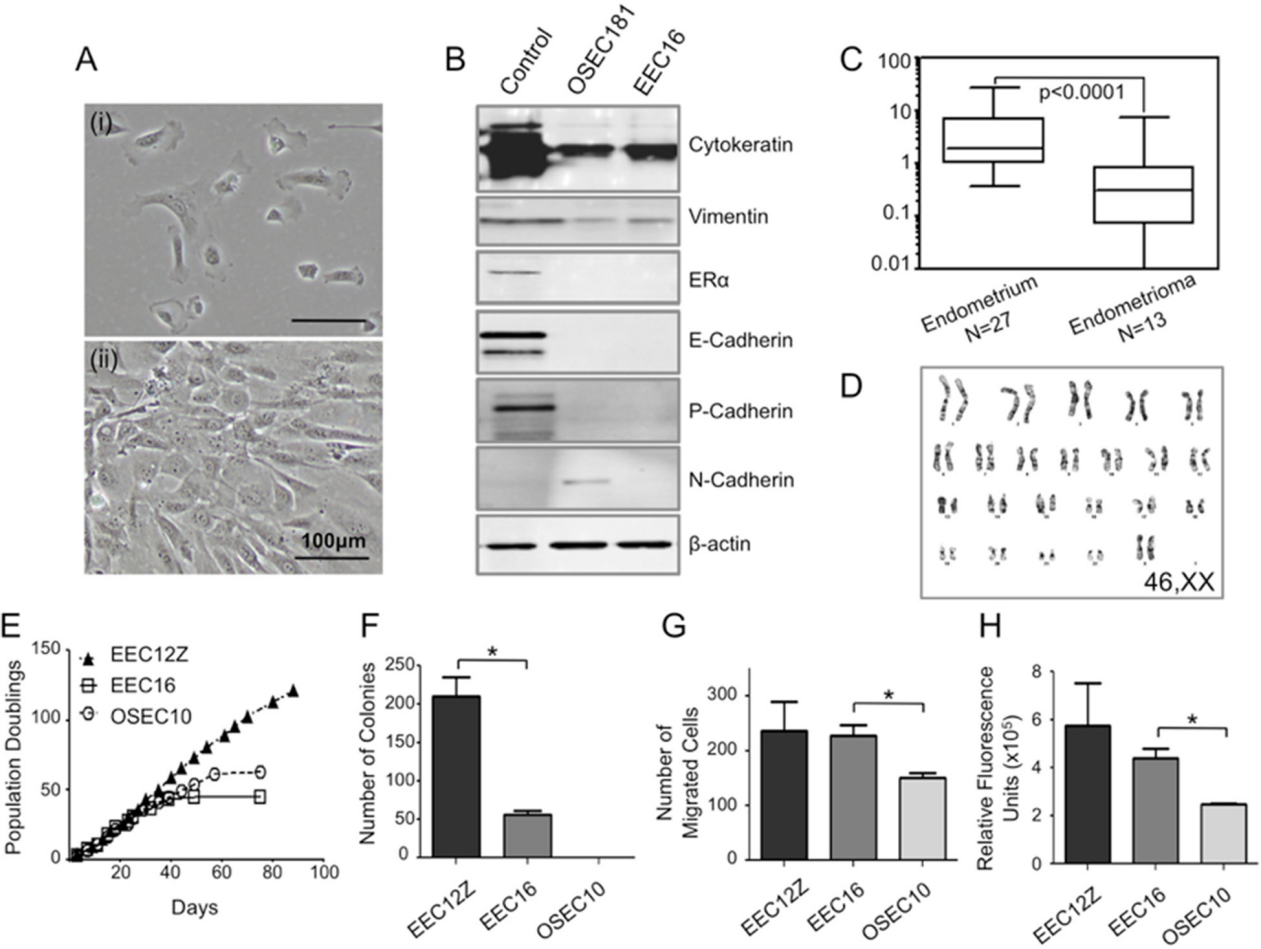
***In vitro *characterization of a novel primary ovarian endometriosis epithelial cell line, EEC16. (A)** EEC16 cells have a mesenchymal-type epithelial morphology *in vitro* at (i) low cell density and (ii) high seeding density. **(B)** Western blot analysis of marker expression. The EEC16 line expressed cytokeratin and vimentin. EEC16 does not express ERα, E-, P-, or N-Cadherin. Beta-actin was used as a loading control. Control lysates used were breast and ovarian cancer cell lines: MCF7, for cytokeratin; T47D, for ERα and P-Cadherin; MDA-MB-231 for vimentin; BT549 for N-Cadherin; and IGROV for E-Cadherin. The difference in ovarian surface epithelial cell (OSEC181) and EEC16 profiles indicates that the EEC16 line is unlikely to be contaminated with normal OSECs. **(C)** Real-time PCR analysis of primary endometrioma and endometrial tissues, *CDH1* is downregulated in endometrioma tissues compared to eutopic endometrium. Expression of *CDH1* in endometrium of women without endometriosis is independent on the stage of the menstrual cycle (Additional file 2: Figure S1). **(D)** The EEC16 line has a normal, female karyotype. **(E)** Growth curves. The EEC16 and OSEC10 lines have a finite *in vitro* lifespan. In comparison, the SV40T-expressing EEC12Z endometriosis line, which has spontaneously acquired the ability to evade replicative crisis, did not show any signs of senescence after extended time in culture [17]. **(F)** In anchorage-independent growth assays the EEC12Z line forms significantly more colonies than EEC16. EEC16 formed colonies in agar up until passage 11. OSEC10 fails to form colonies in soft agar. EEC16 cells are significantly more **(G)** migratory and **(H)** invasive than OSEC10. Each assay was performed three times. * *P* > 0.05, two-tailed unpaired T-test.

We established an endometriosis epithelial cell line (EEC16) from an ovarian endometriosis lesion in a patient with severe endometriosis. Cells displayed an epithelial morphology with mesenchymal characteristics (Figure 1A). We evaluated the expression of several biomarkers and found that EEC16 expressed cytokeratin and vimentin, but did not express N-Cadherin, ERα or P-Cadherin (Figure 1B). Unexpectedly, EEC16 did also not express E-Cadherin, and so we analyzed expression of the *CDH1* gene in primary human ovarian endometriosis tissues and normal endometrial biopsies. We observed that *CDH1* gene expression is significantly lower in human ovarian endometriosis tissues compared to eutopic endometrium (in eutopic endometrium, *CDH1* expression is independent on the cycle phase, Additional file 2: Figure S1), which suggests the lack of E-Cadherin expression by EEC16 is not atypical for ovarian endometriosis (Figure 1C). The EEC16 line was karyotypically normal (46,XX) (Figure 1D). Critically, EEC16 biomarker expression differed from that of a normal ovarian surface epithelial cell line harvested from the ovary of a woman unaffected by endometriosis and so was not the result of outgrowth of contaminating ovarian epithelial cells.

The *in vitro* phenotype differed significantly between EEC16 and normal ovarian surface epithelial cells (OSECs). We also compared the phenotype of this newly established EEC16 line to a previously described epithelial cell line generated from a peritoneal endometriotic lesion (EEC12Z) and immortalized with the SV40 large T antigen [17]. Typical of normal, primary cells in culture, both EEC16 and OSEC cultures had a limited *in vitro* lifespan (Figure 1E). By contrast, the immortalized EEC12Z line did not show any signs of crisis or senescence even after extended passaging in culture (Figure 1E) [17]. Unlike OSECs, EEC16 cultures exhibited phenotypes typically associated with neoplastic transformation. EEC16 formed colonies in anchorage independent growth assays (Figure 1F). Colonies formed by EEC16 in the anchorage-independent growth assays were fewer in number than those formed by EEC12Z (unpaired T-test, P > 0.05), suggesting EEC16 has a less transformed phenotype than EEC12Z. However, the EEC16 line was more migratory and invasive compared to normal OSECs (unpaired T-test, P > 0.05, Figure 1 G&H) but did not differ from EEC12Z in these characteristics. EEC16 was non-transformed *in vivo* and did not reproducibly form lesions when xenografted into nude mice (data not shown). Overall, EEC16 and EEC12Z lines show morphological, phenotypic and molecular characteristics that reflect features typical of human endometriosis lesions and have a more transformed phenotype *in vitro* than OSEC cells.

### Whole transcriptome analysis of EEC16

**Figure 2:**
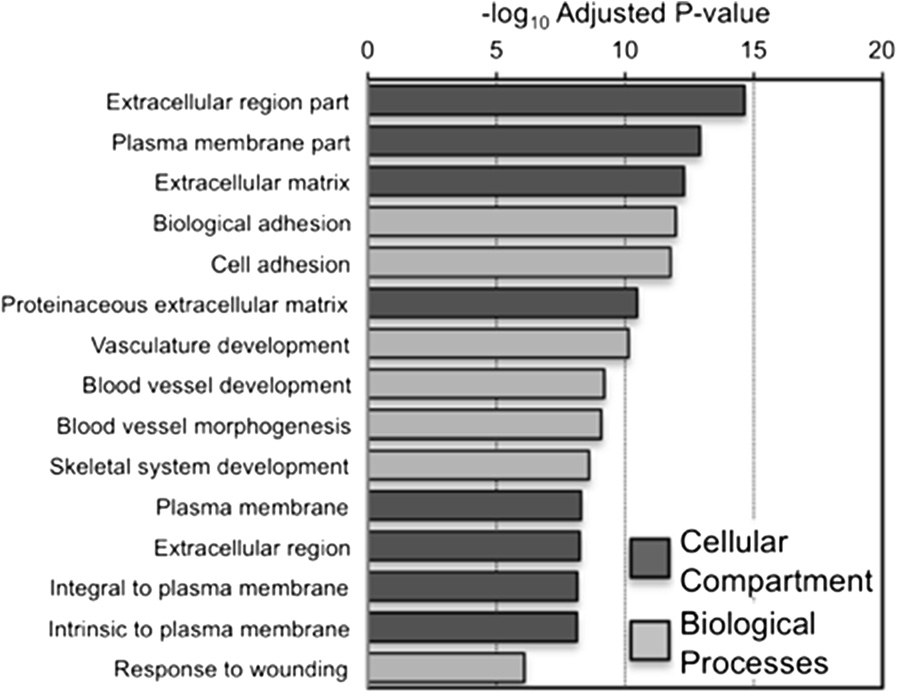
**RNAseq analysis of the transcriptome of EEC16.** Gene ontological analyses of all genes significantly differentially expressed between EEC16 and OSEC11, performed using DAVID (http://david.abcc.ncifcrf.gov) with Benjamini adjusted p-values.

We performed RNA-sequencing to compare the transcriptome between primary EEC16 and OSEC lines. There were 1780 genes significantly differentially expressed between the two cell lines (q value <0.05). The top differentially expressed genes are listed in Table 1. Genes that were expressed more highly in EEC16 included hyaluronan synthase 1, keratin 19, cadherin 20 and genes of the aldehyde dehydrogenase 1 family; genes expressed at lower levels in EEC16 included homeobox C11 and C12, renin, superoxide dismutase 3, and calcitonin receptor. Gene ontology analysis showed that the EEC16 transcriptome was significantly enriched for genes expressed in the extracellular milieu compared to OSECs (Benjamini adjusted p = 2.29 × 10^-15^). The most significantly enriched biological processes were adhesion (adjusted p = 1.08 × 10^-11^) and vasculature development (adjusted p = 6.49 × 10^-10^) (Figure 2). We also observed significant enrichment of genes associated with migration and cell contractility, inflammatory responses, and responses to hypoxia (Additional file 3: Table S2).

**Table 1:** RNA sequencing shows significant differences in transcriptomes of the EEC16 and OSEC lines

**Gene symbol**	**Gene name**	**OSEC11 expression**	**EEC16 expression**	**Fold change**	**q-value**
***Highly expressed in EEC16***					
HAS1	Hyaluronan synthase 1	0.19	148.84	777.83	0.00E + 00
NCAM1	Neural cell adhesion molecule 1	0.02	11.33	524.29	3.44E-08
CHL1	Cell adhesion molecule with homology to L1CAM	0.06	29.64	491.01	0.00E + 00
KRT19	Keratin 19	0.74	253.34	341.72	0.00E + 00
PAQR5,Y_RNA	Progestin and adipoQ receptor family member V	0.34	101.55	301.90	3.07E-03
ODZ2	Odz, odd Oz/ten-m homolog 2 (Drosophila)	0.05	13.68	288.70	1.19E-06
FBLN2	Fibulin2	0.44	108.01	246.49	4.84E-11
SLC24A3	Solute carrier family 24 (sodium/potassium/calcium exchanger), member 31	0.02	4.47	244.08	4.34E-06
CDH20	Cadherin 20, type 2	0.05	12.94	242.76	5.11E-07
MST1R	Macrophage stimulating 1 receptor (c-met-related tyrosine kinase)	0.02	4.09	238.53	2.60E-03
SLITRK5	SLIT and NTRK-like family, member 5	0.02	3.76	236.12	4.86E-06
KRT19P1	Keratin 19 pseudogene 1	0.07	15.32	222.27	6.83E-06
CATSPER2	Cation channel, sperm associated 2	0.01	2.39	172.42	1.99E-04
SLC35F3	Solute carrier family 35, member F3	0.03	3.89	147.18	6.17E-03
CCDC85A	Coiled-coil domain containing 85A	0.04	5.32	145.01	1.46E-03
ALDH1A2	Aldehyde dehydrogenase 1 family, member A2	0.16	21.60	137.26	0.00E + 00
NOVA1	Neuro-oncological ventral antigen 1	0.04	4.50	123.52	6.61E-09
NEBL	Nebulette, Actin-binding Z-disk protein	0.22	26.32	122.11	3.89E-13
AC121334.1,KIF21A	Kinesin family member 21A	7.08	804.71	113.71	6.09E-04
ST6GALNAC3	ST6 (alpha-N-acetyl-neuraminyl-2,3-beta-galactosyl-1,3)-N-acetylgalactosaminide alpha-2,6-sialyltransferase 3	0.02	2.17	107.18	6.71E-08
PMS2	PMS2 postmeiotic segregation increased 2 (S. cerevisiae)	4.99	524.34	105.00	1.84E-05
MMEL1	Membrane metallo-endopeptidase-like 1	0.04	4.06	99.76	1.19E-02
SULT1E1	Sulfotransferase family 1E, estrogen-preferring, member 1	0.19	18.17	94.01	2.70E-05
PTPRD	Protein tyrosine phosphatase, receptor type, D	0.03	2.64	90.18	2.44E-03
FBN2	Fibrillin 2	0.43	36.94	86.48	0.00E + 00
DIRAS3	DIRAS family, GTP-binding RAS-like 3	3.47	281.89	81.34	0.00E + 00
DSC2	Desmocollin 2	0.06	4.98	80.65	1.02E-09
CD7	CD7 molecule, T-cell leukemia antigen	0.78	62.57	80.60	1.34E-11
RSPO4	R-spondin 4	0.03	2.29	78.55	1.92E-02
C3AR1	Complement component 3a receptor 1	0.07	5.56	77.19	5.05E-05
***Highly expressed in OSEC11***					
H19,MIR675	H19, imprinted maternally expressed transcript (non-protein coding); microRNA675	79.38	0	*NA*	8.04E-12
AR	Androgen receptor	4.16	0	*NA*	2.46E-09
CALCR	Calcitonin receptor	3.19	0	*NA*	3.37E-06
DCSTAMP	Dendrocyte expressed seven transmembrane protein	3.55	0	*NA*	1.37E-04
REN	Renin	113.02	0	*NA*	2.81E-04
MGP	Matrix Gla protein	56.34	0	*NA*	3.77E-04
KISS1	KiSS-1 metastasis-suppressor	98.79	0	*NA*	9.30E-04
RP11-13 L2.4		7.26	0	*NA*	1.21E-03
LEF1	Lymphoid enhancer-binding factor 1	4.58	0	*NA*	1.46E-03
HOXC12	Homeobox C12	9.19	0	*NA*	1.91E-03
DACH2	Dachshund homolog 2 (Drosophila)	2.62	0	*NA*	2.05E-03
LPL	Lipoprotein lipase	0.88	0	*NA*	2.08E-03
WNT16	Wingless-type MMTV integration site family, member 16	2	0	*NA*	2.60E-03
PPP1R14A	Protein phosphatase 1, regulatory (inhibitor) subunit 14A	10.01	0	*NA*	6.06E-03
WFDC1	WAP four-disulfide core domain 1	2.49	0	*NA*	6.57E-03
SOD3	Superoxide dismutase 3, extracellular	11.15	0	*NA*	6.65E-03
GSTM5	Glutathione S-transferase mu 5	2.37	0	*NA*	8.57E-03
MKRN3	Makorin ring finger protein 3	2.24	0	*NA*	9.50E-03
LINC00460	Long intergenic non-protein coding RNA 460	3.24	0	*NA*	9.83E-03
C8orf4	Chromosome 8 open reading frame 4	6.8	0	*NA*	1.20E-02
PPAPDC3	Phosphatidic acid phosphatase type 2 domain containing 3	3.84	0	*NA*	1.27E-02
LYPD1	LY6/PLAUR domain containing 1	4.58	0	*NA*	1.50E-02
RCSD1, RP3-455 J7.4	RCSD domain containing 1	1.64	0	*NA*	1.51E-02
ITIH3	Inter-alpha-trypsin inhibitor heavy chain 3	2.11	0	*NA*	1.59E-02
MAOB	Monoamine oxidase B	1.13	0	*NA*	1.69E-02
HOXC11	Homeobox C11	1.68	0	*NA*	1.78E-02
ITPRIPL1	Inositol 1,4,5-trisphosphate receptor interacting protein-like 1	1.21	0	*NA*	1.96E-02
CD36	CD36 molecule (thrombospondin receptor)	1.88	0	*NA*	2.26E-02
KIAA1456	KIAA1456	1.59	0	*NA*	2.28E-02
IQGAP2	IQ motif containing GTPase activating protein 2	1.24	0	*NA*	2.46E-02

As shown by whole transcriptome RNA sequencing, 1780 genes are differentially expressed between primary EEC16 and OSEC lines. The 60 most significantly differentially expressed genes are listed here.

### Establishing three-dimensional models of human endometriosis

**Figure 3:**
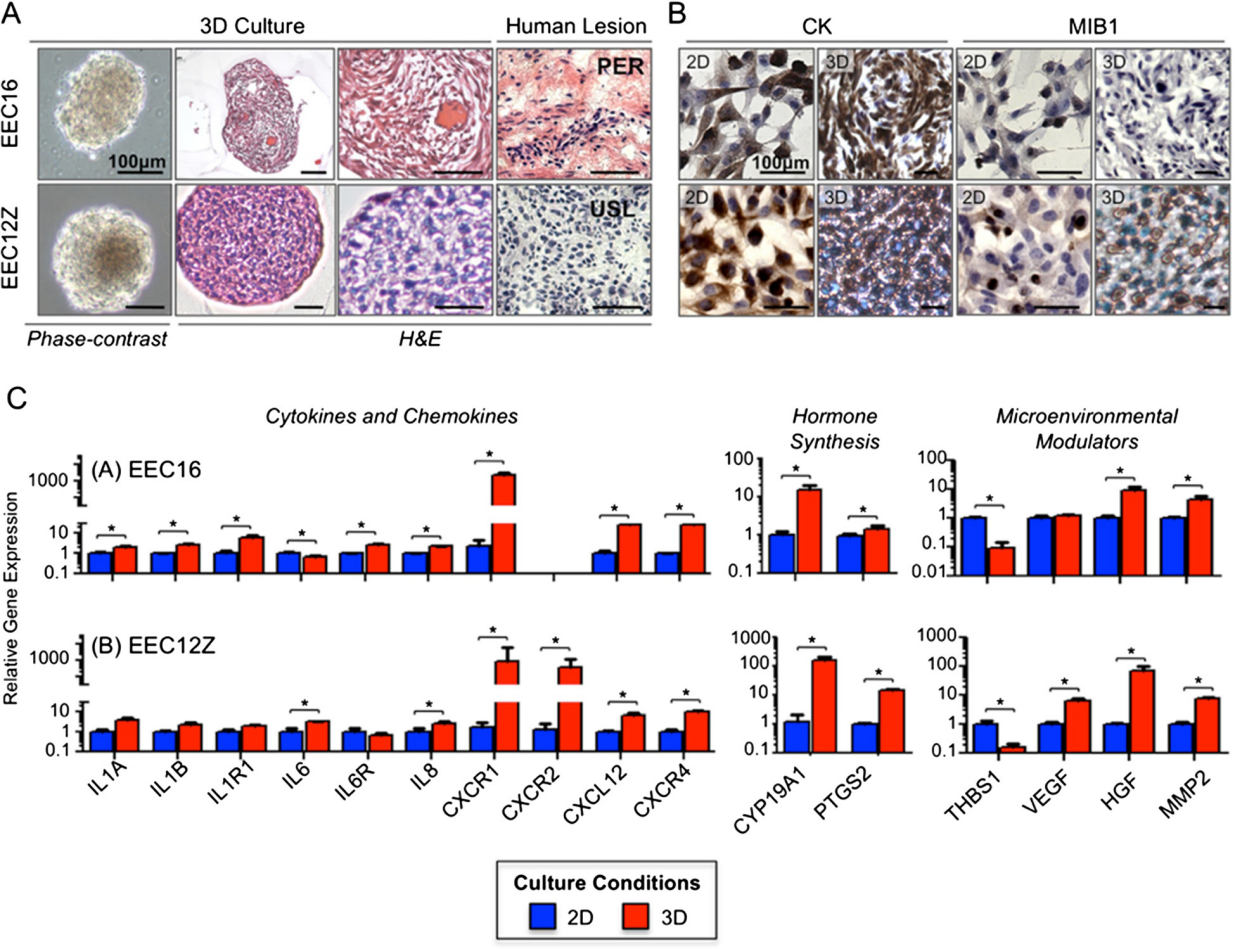
**Histological and molecular characterization of 3D EEC models. (A)** After 7 days of 3D culture, EEC12Z and −16 both form dense, smooth and symmetrical spheroids. H&E staining indicates that the spheroids are highly cellular. EEC spheroids are similar to human endometriosis lesions in the uterosacral ligament (USL) and the peritoneum (PER). **(B)** Immunohistochemical staining of EECs in 3D. Cytokeratin expression is increased in 3D versus 2D. For EEC16, MIB1 expression decreased in 3D compared to 2D. In EEC12Z, MIB1 expression in increased in 3D versus 2D. **(C)** Expression of genes relevant in endometriosis in EEC16 and EEC12Z after culture in 3d for 7 days. *P > 0.05, Two-Tailed Student’s T-Test. Expression is displayed relative to the expression of each gene in 2D. Expression of EGF and FGF9 was also examined and showed no significant change in 2D and 3D cultured EECs (data not shown).

We established EEC16 and EEC12Z as *in vitro* 3D models by culturing cells in non-adherent conditions using polyHEMA-coated cell culture plastics. Both EEC16 and EEC12Z lines began to aggregate within 24 hours and formed smooth, symmetrical spheroid structures (Figure 3A). After 7 days of culturing EEC16 spheroids measured 79.3 ± 15.5 μm in diameter. EEC12Z spheroids were significantly larger in size, measuring 225.7 ± 23.7 μm in diameter (unpaired T-test, P > 0.05). The histological and molecular features of the 3D EEC models were compared with primary human endometriotic lesions. Analysis of hematoxylin and eosin stained sections showed that EEC spheroids were highly cellular and bore histological similarities to human endometriosis tissues such as lesions in the uterosacral ligament and in the peritoneum (Figure 3A). Immunohistochemical staining revealed that 100% of EEC 16 and 12 expressed cytokeratin. Staining intensity for cytokeratin was increased in cells grown in 3D compared with 2D. Finally, 3D cultures of EEC16 had lower proliferative indices compared to the same cells cultured in 2D (17.1% ± 0.7% versus 2.8% ± 0.4%), whereas EEC12Z in 2D had lower proliferative indices than 3D culture counterparts (8.1% ± 1% versus 33% ± 2%) (Figure 3B).

### Candidate gene expression analysis of 3D cultured EEC16

We used semi-quantitative real-time PCR (qPCR) to analyze changes in the expression of genes relevant to endometriosis biology when EEC cultures were transitioned from a 2D to 3D microenvironment (Figure 3C). We focused on genes found in pathways that are involved in immune responses (such as interleukins), microenvironmental interactions (e.g. matrix metalloproteinases and growth factors) and hormonal signaling. Trends in gene expression were highly similar in the two cell line models. Several chemokines, interleukins and their receptors were significantly upregulated in 3D compared to 2D, but particularly *IL6*, *IL8* and its receptor *CXCR1*, and *CXCL12* and its receptor *CXCR4*. The microenvironmental modulators hepatocyte growth factor (*HGF*) and matrix metalloproteinase 2 (*MMP2*) were also significantly upregulated in 3D cultures. Expression of genes involved in the production of prostaglandin (cyclooxygenase 2, *PTGS2*) and estrogen (aromatase, *CYP19A1*) also tended to increase in 3D cultures. Significant downregulation of thrombospondin-1 (*TSP-1*), an inhibitor for neovascularization, was observed in both cell lines and vascular endothelial growth factor, a pro-angiogenic signaling protein, was upregulated in 3D cultures of EEC12Z, the net effect being that pro-angiogenic signaling is enhanced in 3D cultured EECs. Thus, 3D cultures exhibit gene expression profiles that are similar to human endometriosis, while many transcriptomic hallmarks of EMS are reduced/lost when EEC lines are cultured in 2D.

## Discussion

Endometriosis is a common benign gynecological disease, with many clinical consequences for the affected patient such as infertility, chronic pain and a higher risk of ovarian cancer. There is both a basic research and clinical need for better *in vitro* endometriosis models to help understand the underlying biology and etiology of the disease and to identify novel therapeutic targets.

In this study, we describe establishing a novel cell culture model of ovarian endometriosis, EEC16. One challenge when culturing ovarian endometriosis tissues is avoiding contamination by stromal cells or normal adjacent ovarian epithelia. After isolation and culture, 100% of EEC16 cells expressed cytokeratins indicating the culture is epithelial in origin. Furthermore, in contrast to normal OSECs, EEC16 did not express N-Cadherin, and RNA-sequencing profiles showed a 342-fold upregulation of an endometriosis marker, keratin 19, in EEC16 compared to OSECs [19]. This suggests that EEC16 represents an uncontaminated culture of primary ovarian endometriosis epithelial cells. It is known that within endometriosis lesions heterogeneous epithelial cell populations exist. The EEC16 line appears to represent the subpopulation of cells that lack E-cadherin expression and are more invasive in *vitro*[17,20]. Consistent with this, EEC16 expressed vimentin, but not E-cadherin, was invasive and exhibited a partially transformed phenotype in *in vitro* assays. This is in contrast to the phenotype of other primary cells including OSECs, human mammary epithelial cells and fallopian tube epithelial cells [14]. While the novel EEC16 culture maintained expression of the majority of endometriosis markers we tested, expression of ERα was lost. Loss of steroid hormone receptor expression is a common in cultured endometriosis samples and this limitation can be easily circumvented by artificially overexpressing this gene [21]. The RNAseq analysis identified many genes that distinguished EEC16 and OSEC11; we propose that these genes represent novel candidate endometriosis biomarkers and/or novel drivers of endometriosis. For example expression of *H19*, a well known, imprinted, long-non coding RNA [22,23], was high in OSEC11 but absent in EEC16, which may suggest a role for *H19* in endometriosis development. Conversely, adhesion molecules highly expressed by EEC16 (*NCAM* and *CHL1*) but showing only minimal expression in OSEC11 may perhaps be involved in the implantation of endometriosis epithelial cells onto the peritoneum and ovary. Alternatively, genes that distinguish EEC16 and OSEC11 may simply reflect normal differences between cells of ovarian and endometrial origin. Further work will be required using normal endometrial cells as a control to confirm whether the identified genes are involved in the development of endometriosis but nonetheless, a number of the candidate genes we identified warrant further study in *in vitro* and *in vivo* models of endometriosis, as well as in primary tissues.

The geometry, elasticity and tensile forces of a tissue, as well as cell-cell/cell-matrix interactions, can all influence the cellular phenotype but these factors are absent in traditional monolayer cultures. To our knowledge, this is the first report of 3D *in vitro* modeling of endometriosis as spheroids. Histologically, EEC16 spheroids were highly reminiscent of peritoneal lesions. EEC16 was from a lesion located on the ovarian surface, and we note that our observations are consistent with previous reports that find ovarian surface lesions, upon histological examination, resemble peritoneal lesions more closely than cystic endometriomas within the ovarian cortex [2]. A striking feature of the 3D endometriosis models was the close resemblance of human endometriosis lesions on a molecular level. Culturing cells in a 3D environment lead to changes in the expression of genes involved in pathophysiologic pathways responsible for the formation and growth of endometriosis lesions as well as for endometriosis related symptoms in patients. An important clinical need could be met by using these models to develop novel treatments targeting pathways such as cytokine and interleukin signaling, cellular prostaglandin and estrogen biosynthesis, growth factor and neovascularization signaling. For instance, 3D models of endometriosis could be used to perform high-throughput *in vitro* screens to identify novel small molecule inhibitor therapies for endometriosis. These highly specific drugs would potentially have the advantage of far fewer unwanted side effects than current treatment regimens.

Finally, epidemiological and histopathological studies reported that endometriosis patients have an elevated risk of developing ovarian cancers with a clear cell and endometrioid histology [3,5,6]. Numerous genes, including *ARID1A* and *WNT4*[24-29] have been implicated in the development of endometriosis-associated ovarian cancer. 3D models of endometriosis could now be used to study the functional role of these specific genes during tumorigenesis and to model the stepwise development from endometriosis precursor lesions to ovarian cancer.

## Conclusions

Our overall conclusion is that 3D models of endometriosis are superior to existing monolayer culture techniques. It is clear that these 3D models will have diverse applications for endometriosis and ovarian cancer research. Improved understanding of the biological links between endometriosis and ovarian cancer could help to predict which endometriosis lesions are most susceptible to neoplastic transformation. In such cases women could be offered preventive surgery, intensive screening or perhaps chemoprevention. Moreover, we and others find that 2D and 3D *in vitro* models of malignant cells show differential responses to therapeutic agents [15,30]; since endometriosis 3D models more closely resemble the *in vivo* microenvironment of endometriosis, the potential for identifying and translating novel targeted therapeutic strategies will be greatly enhanced by using these models.

## Abbreviations

2D: Three-dimensional; 3D: Three-dimensional; EEC: Endometriosis epithelial cell; GO: Gene ontology; OSEC: Ovarian surface epithelial cells; PCR: Polymerase chain reaction; RNA: Ribonucleic acid; USC: University of Southern California.

## Competing interests

All authors declare that they have no financial or non-financial competing interests.

## Authors’ contributions

DB, KL and SAG designed the study and wrote the manuscript. DB and KL conducted and analyzed the experiments. CT collected human endometriosis tissue samples and PNR performed the karyotyping. ASP created the EEC12Z cell line used in this study. All authors contributed to and approved the final draft of the manuscript.

## Supplementary Material

Additional file 1: Table S1 Primer sequences for gene expression analysis. PCR primers were designed using Primer3 (http://primer3.sourceforge.net/) or were identified using the Harvard primer bank (http://pga.mgh.harvard.edu/primerbank/). Click here for file

Additional file 2: Figure S1 E-Cadherin expression in the endometrium during the menstrual cycle. E-cadherin expression in the endometrium of women (not affected by endometriosis) is not influenced by stage of the menstrual cycle. Click here for file

Additional file 3: Table S2 Gene ontology analyses of the EEC16 transcriptome. We performed a gene ontology analysis of the genes highly expressed in EEC16 cells compared to OSEC 11. We detected a significant enrichment of genes associated with relevant biological processes in endometriosis were found such as adhesion, vasculature development migration, cell contractility, inflammatory responses, and responses to hypoxia. Click here for file
