# Supplementary figures and images for: Novel three-dimensional in vitro models of ovarian endometriosis
Source: J Ovarian Res. 2014 Feb 6;7:17. doi: 10.1186/1757-2215-7-17 (PMC4015880; doi:10.1186/1757-2215-7-17)

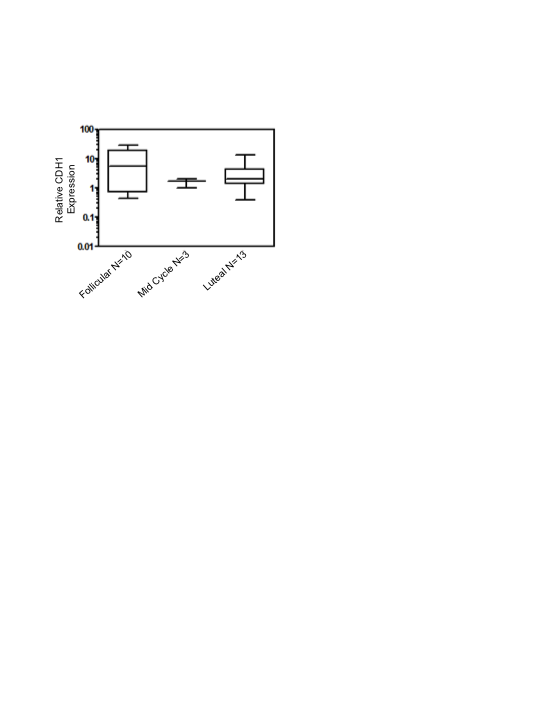

Supplement: Additional file 2: Figure S1 — E-Cadherin expression in the endometrium during the menstrual cycle. E-cadherin expression in the endometrium of women (not affected by endometriosis) is not influenced by stage of the menstrual cycle. [file 1757-2215-7-17-S2.png]
